# Comparing Neurocognitive Profile of Patients with Borderline Personality and Bipolar–II Disorders

**Published:** 2019-04

**Authors:** Valiolah Akbari, Parvin Rahmatinejad, Seyed Davood Mohammadi

**Affiliations:** 1 Department of Psychiatry, Qom University of Medical Sciences, Qom, Iran.; 2 Clinical Psychology, Forghani Hospital Research Development Committee, Qom University of Medical Sciences, Qom, Iran.; 3 Clinical Psychology, Department of Psychiatry, School of Medicine, Fasa University of Medical Sciences, Fasa, Iran.

**Keywords:** *Bipolar Disorder*, *Borderline Personality Disorder*, *Neurocognitive Profile*

## Abstract

**Objective:** The present study was conducted to compare neurocognitive profile in patients with borderline personality disorder (BPD) and bipolar II disorder (BD-II) and to find whether BPD can be classified as one of bipolar spectrum disorders.

**Method** **:** A total of 35 patients with BPD and 35 euthymic patients with BDII disorder were selected by convenience sampling method. These 2 groups were compared with 30 healthy individuals using neurocognitive battery tests that assessed cognitive flexibility and set-shifting, response inhibition, problem-solving, decision-making, and sustained and selective attention. Data were analyzed using independent t test, X2 and ANOVA.

**Results: **Patients with euthymic BDII and BPD had poorer performance than the healthy group in most neurocognitive domains (p<0.05). Both patient groups showed similar functions in cognitive flexibility and set-shifting, decision-making, sustained and selective attention, and problem-solving (p<0.05). BPD patients had more elevated response inhibition deficits than BD-II patients (P<0.05). Also, BPD patients had poorer performance in planning compared to BD-II patients (P<0.05).

**Conclusion: **The results provided empirical support for previous findings which have reported that patients with BPD and BD-II show neurocognitive dysfunctions. Despite the similarity between these 2 clinical groups in terms of neurocognitive profile in this study, more extensive studies are needed to confirm the hypothesis that BPD can be conceptualized as one of bipolar spectrum disorders.

Precise distinction between borderline personality disorder (BPD) and bipolar disorders (BD) is one of the main challenges among clinical therapists and psychopathologists (1, 2). Bipolar disorders- particularly bipolar II- and BPD have extensive overlap in such symptoms as impulsivity and instability (3, 4). Evidence of a high comorbidity between these two disorders (5), extensive overlap in symptoms (3, 4), and (BD-II) similarity in phenomenology and treatment response (6) have made some investigators to assert that BPD is part of bipolar spectrum disorders (7, 8). While the results of some studies show that BPD does not fall within the bipolar spectrum (9, 10, 11), other studies (3, 7, 12) have provided some evidence that tend to confirm this hypothesis. Neurocognitive studies may help investigators to provide greater insight into the relationship between these 2

 Conditions through identifying neurocognitive functions that are similar or different in the 2 disorders. Results of many studies that had often been conducted separately, showed that neurocognitive function is impaired in patients with BPD and bipolar spectrum disorder. 

One study showed that attention, memory, and executive function are cognitive domains that were mostly impaired in patients with BD (13). A meta-analysis of neuropsychological studies of patients with BD indicated that patients who were tested during a manic/mixed or depressed phase of illness showed severe impairment on measures of verbal learning, and patients who were tested during depressed phase showed severe decrease on measures of phonemic fluency (14). 

compared to healthy controls. However, there were no significant difference between the 2 subtypes (15).

One research reported that patients with bipolar I and II showed impairment in cognitive domains, such as inhibition, set-shifting, verbal fluency, and visual recall, One systematic review and meta-analysis in patients with BD (type I or II) and healthy controls identified 6 domains of executive function, including set-shifting, inhibition, planning, verbal fluency, working memory, and attention. In general, results showed that patients with BD-I performed worse than healthy group and patients with BD-II demonstrated impairment in verbal fluency, working memory, set-shifting, and attention (16). 

 Research studies indicated that BPD was characterized by deficits in nonverbal executive function and nonverbal memory (17), recovery processes of the immediate and differed memory, working memory, sustained attention and processing rate, verbal fluency, impulse control, cognitive flexibility, abstraction and planning (18), working memory, perseveration, decision-making (19), and feedback evaluation (20). 

Studies that were conducted separately found bipolar and BPD to be associated with multiple abnormalities in prefrontal attentional networks and mechanisms of inhibitory control which may contribute to impulsivity and poor affect regulation (21). However, to date, no literature has directly compared bipolar spectrum disorders and BPD patients according to neurocognitive profile using the same methodological approaches. Thus, the goal of this study was to examine the relationship between BD-II and BPD based on neuropsychological profile to clarify diagnostic and classification issues. Also, understanding the differences and similarities of neurocognitive profile in BPD and BD-II has a significant therapeutic implication. It can help therapists to use the best and most appropriate psychotherapy, pharmacotherapy, and rehabilitation strategies for both patients.

## Materials and Methods

In this study, 35 patients with BD-II and 35 with BPD were consecutively selected from the outpatient population of Kamkar-Arabnia hospital in Qom, Iran, using the following inclusion criteria: age 18-65 years, diagnosis of BD-II or BPD according to SCID (22) and SCID-II (23), being euthymic in BD-II (defined by the Persian version of the Hamilton Rating Scale for Depression (HRSD) ≤7, (24) and Young Mania Rating Scale (YMRS) ≤12 (25)) for at least 3 months. The exclusion criteria were as follow: recent or lifetime history of substance abuse, head injury, epilepsy, mental retardation (IQ<70), neurological diseases, treatment with ECT in the last 2 years. In addition, a control group were recruited from the hospital staff and matched with the patient groups by age, sex, and years of education. 

The researcher provided a detailed description of how the test was going to be performed for each participant prior to testing. Also, the participants were entitled to leave the test at any time if they decided to leave the study. Moreover, participants were assured about the anonymity and confidentiality of the information. Ethical code was obtained from Ethics Committee of Qom University of Medical Sciences (IR.MUQ.REC.021). 

***Neurocognitive Assessment***

Patients completed neurocognitive battery tests: (1) Wisconsin Card Sorting Test (WCST; (26)), which evaluates cognitive flexibility and set-shifting; (2) Iowa Gambling Task (IGT; (27)), which assesses decision-making; (4) Stroop Color-Word Interference Test (SCWT; (28)), which assesses the ability to response inhibition; (5) Tower of London (ToL; (29)), which is a problem-solving task and detects deficits in planning; (6) Continuous Performance Test (CPT;(30)), which measures sustained and selective attention; and (7) Vocabulary subtest (Wechsler Adult Intelligence Scale (WAIS; (31)), which estimates IQ. All neuropsychological tests were administered by a trained clinical psychologist based on standardized order. In this study, researchers used a computer-administered version of the tests, except for Wechsler Adult Intelligence Scale vocabulary subtest, in which the participants were asked the meaning of 40 words by examiners.

***Statistical Analysis***

Data were analyzed using by SPSS 21.0. The 3 groups were compared on demographical and clinical variables using ANOVA and x^2 test. Neurocognitive variables were normally distributed as assessed by the Kolmogorov-Smirnov test. To decrease the risk of type I errors, ANOVA was conducted with all neurocognitive variables as dependent variables and groups as factor. As neurocognitive tests are naturally correlated, this procedure was considered superior to the Bonferroni inequality correction that would increase type II errors. When significant main effects were present, Tukey post hoc test was used to clarify group effects. Also, the effect sizes (Cohen d) were calculated to find the difference between the 3 groups. Pearson correlation coefficients and Spearman correlation (in nonparametric variables) were used to test the associations between clinical-demographic and neurocognitive variables.

## Results

Clinical-demographic variables of participants are summarized in Table 1. The analysis of data showed that there was no correlation between neurocognitive tests and clinical- demographic characteristics (all p > 0.05). 

According to Table 2, ANOVA analysis indicated significant differences between the groups. Post hoc comparisons showed that BDII and BPD groups both had poorer performance on most domains, such as cognitive flexibility and set-shifting, response inhibition, decision-making, and sustained and selective attention than the healthy group. Generally, BD-II and BPD groups did not show differences in any of the neurocognitive measures assessed. However, BPD group had a trend to perform more poorly than the BD-II groupon errors task on TOL (P = 0.023) and commission task on CPT (P = 0.41). 

**Table 1:** Clinical-Demographic Characteristics of Borderline Personality Disorder, Bipolar II Disorder Patients and Control Group

	**BPD (35)**	**BD-II (35)**	**Healthy ** **controls (30)**	**Test**		**Tukey Post** **Hoc**
Age (mean± S.D)	24.00 ±1.88	28/8±2.44	25.98±2.76	F=.298	P=0.183	
Sex, n (%)						
male	15(42.85)	18(51.43)	18(60)	X^2^=0.456	P=0.434	
female	20(57.15)	17 (48.57)	12(40)			
Education (y, mean± S.D)	12.26±1.08	12.12±2.12	13±2.68	F=.354	P=0.474	
Age at onset (y, mean± S.D)	19.23±1.00	24.00±3.42		F=5.89	P=0.004	A>B>C
Length of illness (y, mean± S.D)	5±2.08	6±1.65		F=0.169	P=0.245	
No. of previous admissions (mean± S.D)	2±1.54	1±21		X^2^=1.25	P=0.134	
Medicines, n (%)						
Mood stabilizers	30(85.71)	35(100)		X^2^=1.43	P=0.176	
Antipsychotics	32(91.42)	23(65.71)		X^2^=6.24	P=0.041	A>B>C
Antidepressants	34(97.14)	29(82.85)		X^2^=3.62	P=0.086	
Premorbid IQ (mean± S.D)	109.8 ±8.12	110.20±9.22	112±8.65	F=0.753	P=0.110	

**Table 2:** Neurocognitive Profile of the Patients with Borderline Personality Disorder, Bipolar II Disorder and Control Group

**Variable ** **(measures)**		**Mean** **(SD)**		**test**		**Tukey ** **Post Hoc**	**Cohen ** ***d***		
	**BPD**	**BD-II**	**Healthy ** **group**	**f**	**p**		**A vs. B**	**B vs. C**	**A vs. C**
Cognitive (flexibility WCST) and set-shifting									
No, of categories	3.06 (1.34)	3.92 (1.89)	5.41 (1.2)	8.37	0.001	A=B<C	0.15	0.50	0.24
Perseverative errors	6.31 (2.26)	5.61 (3.66)	1.41 (1.5)	9.94	0/000	A=B>C	1.00	0.49	0.84
Response inhibition (SCWT)									
Interference time	69.11 (88.10)	52.50 (100.65)	36.90 (68.44)	3.211	0.051	A=B>C	0.88	0.03	0.67
Corrects	79.78 (12.43)	81.18 (21.32)	109.80 (1.93)	4.148	0.041	A=B<C	0.85	0.73	0.14
Problem-solving (TOL)									
Total moves	26.58 (11.29)	18.85 (9.92)	19.40 (5.75)	3.024	0.060	A=B=C	0.11	0.03	0.21
Total time	728.52 (481.29)	636.38 (538.05)	442.10 (209.93)	1.203	0.283	A=B=C	0.23	0.32	0.35
Test time	416.31 (226.38)	311.38 (325.94)	256.50 (161.10)	1.522	0.231	A=B=C	0.76	0.61	0.33
Errors	19.21 (12.14)	8.92(9.73)	8.00 (6.58)	5.563	0.004	A>B=C	0.03	0.43	0.36
Total score	23.63 (6.94)	28.30(9.89)	32.30 (2.9)	4.760	0.014	A=B<C	0.26	0.62	0.02
Decision- making (IGT)									
Total reward	3770.59 (506.53)	4653.57 (390.49)	9800.00 (744.31)	4.351	0.015	A=B<C	0.81	0.67	0.24
Total punishment	8160.29 (926.81)	8333.93 (604.20)	3343.18 (1443.39)	6.159	0.031	A=B>C	0.25	0.16	0.09
Total score	1610.39 (531.36)	1319.64 (358.57)	5456.82 (772.09)	6.047	0.036	A=B<C	0.48	0.41	0.20
Sustained and selective attenti on (CPT)									
Omissions	3.84( 3.62)	3.1(3.7)	0.45( 0.52)	4.015	0.025	A=B>C	0.64	0.13	0.24
Commission	2.15 (2.83)	1.60 (1.99)	0.09 (0.30)	3.151	0.053	A>C; B=A,C	0.47	0.27	0.62
Corrects	144.00 ( 5.37)	145.26 (4.84)	149.45( 0.68)	5.231	0.009	A=B>C	0.56	0.49	0.35

## Discussion

The first finding of this study was that patients with BD-II and BPD had poorer performance than the healthy group on most neurocognitive domains. This result agrees with previous studies that showed patients with euthymic BD demonstrate deficits in cognitive functions (32, 33). Based on previous studies, cognitive impairment affects psychosocial functioning in bipolar patients during euthymic phases (33). However, while cognitive impairments in the euthymic period may be subjected to subclinical and subsyndromal symptoms, the disease-related factors, such as severity of symptoms, number of relapses, and use of medication, may be involved in these neurocognitive deficiencies. Therefore, more precise retrospective and futuristic studies are needed in this regard. However, due to high heterogeneity of symptoms in this disorder and its high concurrency with other psychiatric disorders, the accurate assessment of cognitive impairments in borderline personality disorder is also faced with many difficulties.

Also, these results are consistent with previous findings that indicated patients with BPD had impaired cognitive functions (18, 34). 

Both patient groups showed similar functions in cognitive flexibility and set-shifting, response inhibition, decision-making, sustained and selective attention planning, and problem-solving. However, it seems that Patients with BPD had more elevated response inhibition deficits (and as a result more impulsivity) than BD-II patients, as indicated by vast amount of commission errors on CPT. This finding indicates that patients with BPD had more impulsivity than BD-II patients. Direct neurocognitive comparison studies of BPD and BD-II are still limited. However, one study showed patients with BPD are more impulsive than BD-II patients (35). 

Moreover, patients with BPD had more errors on TOL than bipolar patients, indicating that BPD patients had poorer performance in planning compared to BD-II patients. One study reported that BD patients had poorer performance in strategy formation and planning than BPD patients and healthy group. Also, BPD patients showed deficits in planning compared to BD patients and healthy group (36). 

In the present study, patients with BPD and BD-II performed equally compared to control group on total moves, total time, time violation and test time tasks on TOL, which measured planning. These findings are inconsistent with some of the results obtained from previous studies. Beblo et al, using the Tower of Hanoi test, reported that BPD patients required more moves and significantly longer time to accomplish the task (37). Furthermore, 1 study showed that BPD patients needed longer time for resolution of the task, but they did not differ from controls on the number of moves (38). However, studies found deficits in planning in patients with BD (16, 35). 

However, BPD patients had more errors compared to BD-II patients and healthy group in the TOL tasks. Nevertheless, healthy group had higher total score than BD-II and BPD patients. 

These results highlighted previous findings that indicated BPD and BD patients have deficits in problem-solving (36). It seems that apart from the response style to the TOL tasks, BPD and BD-II patients had poorer performance in their planning and problem-solving, which can show similar underlying etiology for these 2 groups for deficit in these tasks. The results of 1 meta-analysis conducted by Ruocco indicated that the largest deficit in executive function of BPD patients was in planning domain (34). 

As programming and problem-solving are among the main areas of the frontal lobe execution, it seems that prefrontal lobe malfunctioning has a role in the phenomenology of cognitive impairment in borderline personality disorder.

Also, Fue et al, in their study, reported reduced prefrontal activation during TOL and verbal fluency task in patients with bipolar depression. Based on these results, the researchers concluded which planning and problem-solving dysfunctions are related to the impairment of the prefrontal cortex in patients with bipolar depression (40). 

The abilities of planning and problem-solving in individuals with BPD and BD-II have not been extensively studied. However, it seems that some defects in problem-solving abilities of these patients lead to impaired function in daily life. For example, 1 study that was performed on women with BPD revealed that inappropriate problem-solving strategies are predictors of parasuicidal behavior in these patients (39). In patients with BD, 1 study indicated that problem-solving and cognitive flexibility intervention could improve functional outcome (41). 

## Limitation

This study had several limitations. First, BPD and BD-II patients were not assessed for the presence of comorbid psychiatric disorders, such as other personality disorders and axis-I disorders. In addition, one of the factors that might have had an impact on our finding was use of psychiatric drugs in BPD and BD-II Patients. Some studies have indicated that psychiatric medicines, such as antipsychotics, can affect cognitive function in patients (42). Another limitation of the present study was that severity of BPD and bipolar-II was not assessed. Thus, it is recommended that future researches assess the severity of symptoms in these two disorders.

## Conclusion

Our findings suggest that may be the same brain performance in borderline and bipolar-II patients leads to similar neurocognitive profiles and deficits. Categorizing BPD as a BD subtype has raised considerable debate, and further studies are needed to better understand the differences and similarities between the two disorders, particularly in cognitive functions.
